# Supplementary material for: A mixed studies systematic review on the health and wellbeing effects, and underlying mechanisms, of online support groups for chronic conditions
Source: Commun Psychol. 2025 Mar 15;3:40. doi: 10.1038/s44271-025-00217-6 (PMC11910600; doi:10.1038/s44271-025-00217-6)
Supplement: Supplementary file 2 — Supplementary Tables [file 44271_2025_217_MOESM2_ESM.pdf]

A mixed studies systematic review on the health and wellbeing effects, and underlying mechanisms, of online support groups for chronic conditions: Supplementary Tables

Table 1: PRISMA Checklist

Section and Topic	Item #	Checklist item	Location where item is reported
**TITLE**			
Title	1	Identify the report as a systematic review.	Title page
**ABSTRACT**			
Abstract	2	See the PRISMA 2020 for Abstracts checklist.	Abstract
**INTRODUCTION**			
Rationale	3	Describe the rationale for the review in the context of existing knowledge.	Introduction
Objectives	4	Provide an explicit statement of the objective(s) or question(s) the review addresses.	"Current Study"
**METHODS**			
Eligibility criteria	5	Specify the inclusion and exclusion criteria for the review and how studies were grouped for the syntheses.	"Eligibility criteria"<br><br>"Synthesis"
Information sources	6	Specify all databases, registers, websites, organisations, reference lists and other sources searched or consulted to identify studies. Specify the date when each source was last searched or consulted.	"Search criteria"
Search strategy	7	Present the full search strategies for all databases, registers and websites, including any filters and limits used.	Supplementary Tables 2-6
Selection process	8	Specify the methods used to decide whether a study met the inclusion criteria of the review, including how many reviewers screened each record and each report retrieved, whether they worked independently, and if applicable, details of automation tools used in the process.	"Study selection"
Data collection process	9	Specify the methods used to collect data from reports, including how many reviewers collected data from each report, whether they worked independently, any processes for obtaining or confirming data from study investigators, and if applicable, details of automation tools used in the process.	"Data extraction"
Data items	10a	List and define all outcomes for which data were sought. Specify whether all results that were compatible with each outcome domain in each study were sought (e.g. for all measures, time points, analyses), and if not, the methods used to decide which results to collect.	"Data extraction"
	10b	List and define all other variables for which data were sought (e.g. participant and intervention characteristics, funding sources). Describe any assumptions made about any missing or unclear information.	"Data extraction"
Study risk of bias assessment	11	Specify the methods used to assess risk of bias in the included studies, including details of the tool(s) used, how many reviewers assessed each study and whether they worked independently, and if applicable, details of automation tools used in the process.	"Quality assessment"
Effect measures	12	Specify for each outcome the effect measure(s) (e.g. risk ratio, mean difference) used in the synthesis or presentation of results.	N/A
Synthesis methods	13a	Describe the processes used to decide which studies were eligible for each synthesis (e.g. tabulating the study intervention characteristics and comparing against the planned groups for each synthesis (item #5)).	"Study selection"
	13b	Describe any methods required to prepare the data for presentation or synthesis, such as handling of missing summary statistics, or data conversions.	"Data extraction and synthesis"
	13c	Describe any methods used to tabulate or visually display results of individual studies and syntheses.	N/A

Section and Topic	Item #	Checklist item	Location where item is reported
	13d	Describe any methods used to synthesize results and provide a rationale for the choice(s). If meta-analysis was performed, describe the model(s), method(s) to identify the presence and extent of statistical heterogeneity, and software package(s) used.	"Data extraction and synthesis"
	13e	Describe any methods used to explore possible causes of heterogeneity among study results (e.g. subgroup analysis, meta-regression).	N/A
	13f	Describe any sensitivity analyses conducted to assess robustness of the synthesized results.	N/A
Reporting bias assessment	14	Describe any methods used to assess risk of bias due to missing results in a synthesis (arising from reporting biases).	N/A
Certainty assessment	15	Describe any methods used to assess certainty (or confidence) in the body of evidence for an outcome.	N/A
**RESULTS**			
Study selection	16a	Describe the results of the search and selection process, from the number of records identified in the search to the number of studies included in the review, ideally using a flow diagram.	Figure 1
	16b	Cite studies that might appear to meet the inclusion criteria, but which were excluded, and explain why they were excluded.	Supplementary Table 7
Study characteristics	17	Cite each included study and present its characteristics.	Table 2
Risk of bias in studies	18	Present assessments of risk of bias for each included study.	Supplementary Tables 8-12
Results of individual studies	19	For all outcomes, present, for each study: (a) summary statistics for each group (where appropriate) and (b) an effect estimate and its precision (e.g. confidence/credible interval), ideally using structured tables or plots.	N/A
Results of syntheses	20a	For each synthesis, briefly summarise the characteristics and risk of bias among contributing studies.	Results
	20b	Present results of all statistical syntheses conducted. If meta-analysis was done, present for each the summary estimate and its precision (e.g. confidence/credible interval) and measures of statistical heterogeneity. If comparing groups, describe the direction of the effect.	N/A
	20c	Present results of all investigations of possible causes of heterogeneity among study results.	N/A
	20d	Present results of all sensitivity analyses conducted to assess the robustness of the synthesized results.	N/A
Reporting biases	21	Present assessments of risk of bias due to missing results (arising from reporting biases) for each synthesis assessed.	N/A
Certainty of evidence	22	Present assessments of certainty (or confidence) in the body of evidence for each outcome assessed.	N/A
**DISCUSSION**			
Discussion	23a	Provide a general interpretation of the results in the context of other evidence.	Discussion
	23b	Discuss any limitations of the evidence included in the review.	"Limitations"
	23c	Discuss any limitations of the review processes used.	"Limitations"
	23d	Discuss implications of the results for practice, policy, and future research.	"Implications"
**OTHER INFORMATION**			
Registration and protocol	24a	Provide registration information for the review, including register name and registration number, or state that the review was not registered.	"Protocol and registration"
	24b	Indicate where the review protocol can be accessed, or state that a protocol was not prepared.	"Protocol and registration"
	24c	Describe and explain any amendments to information provided at registration or in the protocol.	"Protocol and registration"
Support	25	Describe sources of financial or non-financial support for the review, and the role of the funders or sponsors in the review.	Abstract and Acknowledgements

Section and Topic	Item #	Checklist item	Location where item is reported
Competing interests	26	Declare any competing interests of review authors.	“Competing interests”
Availability of data, code and other materials	27	Report which of the following are publicly available and where they can be found: template data collection forms; data extracted from included studies; data used for all analyses; analytic code; any other materials used in the review.	“Data availability”

From: Page MJ, McKenzie JE, Bossuyt PM, Boutron I, Hoffmann TC, Mulrow CD, et al. The PRISMA 2020 statement: an updated guideline for reporting systematic reviews. BMJ 2021;372:n71. doi: 10.1136/bmj.n71. This work is licensed under CC BY 4.0. To view a copy of this license, visit https://creativecommons.org/licenses/by/4.0/

Tables 2-7: Full search strategy

Tables 2-7 display the full search strategy for each database and grey literature search including the number of records identified for each search term. All searches were based on the study population (individuals with a Chronic Condition) and study intervention (online support group).

The online platform Ovid was used to search Embase, Medline and PsycInfo. Free text search items were searched in the abstract and title for Embase, Medline and PsycInfo (represented by '.ab.ti' in the search terms) and in the topic for Web of Science, which included title, abstract, author keywords and keywords plus. Search items beginning with 'exp' refer to database controlled vocabulary identified in relation to the words 'chronic condition' and 'online support group'. Medline did not have controlled vocabulary in relation to 'online support group'.

Table 2: search terms for Embase

Embase 1974 to September 10, 2024		
**Concept**	**Number**	**Query**
Population (Individuals with a Chronic Condition)	1	(chronic adj illness*).ab,ti.
	2	(chronic adj disease*).ab,ti.
	3	(chronic adj condition*).ab,ti.
	4	(chronic adj infection*).ab,ti.
	5	(chronic adj health*).ab,ti.
	6	(chronic adj respiratory*).ab,ti.
	7	(chronic adj neurologic*).ab,ti.
	8	(chronic adj pain*).ab,ti.
	9	(chronic adj fatigue*).ab,ti.
	10	(long term adj2 condition*).ab,ti.
	11	"diabetes".ab,ti.
	12	"hypertension".ab,ti.
	13	"angina".ab,ti.
	14	"cardiovascular disease".ab,ti.
	15	"chronic obstructive pulmonary disease".ab,ti.
	16	"multiple sclerosis".ab,ti.
	17	"arthritis".ab,ti.
	18	"cancer".ab,ti.
	19	"irritable bowel syndrom*".ab,ti.
	20	"myalgic encephalomyelitis".ab,ti.
	21	"CFS".ab,ti.
	22	"ME/CFS".ab,ti.
	23	"long covid".ab,ti.
	24	"coronary heart disease".ab,ti.

25	"cystic fibrosis".ab,ti.
26	"ulcerative colitis".ab,ti.
27	"chronic kidney disease".ab,ti.
28	"haemophilia".ab,ti.
29	"lupus".ab,ti.
30	"HIV".ab,ti.
31	"Crohn's disease".ab,ti.
32	"sickle cell anemia".ab,ti.
33	"fibromyalgia".ab,ti.
34	"inflammatory bowel disease".ab,ti.
35	"systemic sclerosis".ab,ti.
36	exp chronic active hepatitis/
37	exp chronic allograft nephropathy/
38	exp chronic arthritis/
39	exp chronic bronchitis/
40	exp chronic cluster headache/
41	exp chronic constipation/
42	exp chronic cough/
43	exp chronic depression/
44	exp chronic disease/
45	exp chronic fatigue syndrome/
46	exp chronic graft versus host disease/
47	exp chronic granulomatous disease/
48	exp chronic hepatitis B/
49	exp chronic hepatitis C/
50	exp chronic infection/
51	exp chronic inflammation/
52	exp chronic inflammatory pain/

	53	exp chronic intermittent hypoxia/
	54	exp "chronic kidney disease-mineral and bone disorder"/
	55	exp chronic kidney failure/
	56	exp chronic liver disease/
	57	exp chronic liver failure/
	58	exp chronic lung disease/
	59	exp chronic liver failure/
	60	exp chronic low grade inflammation/
	61	exp chronic lung disease/
	62	exp chronic myeloid leukemia/
	63	exp chronic lymphatic leukemia/
	64	exp chronic myelomonocytic leukemia/
	65	exp chronic neutrophilic leukemia/
	66	exp chronic obstructive lung disease/
	67	exp chronic otitis media/
	68	exp chronic pain/
	69	exp chronic pancreatitis/
	70	exp chronic periodontitis/
	71	exp chronic progressive external ophthalmoplegia/
	72	exp chronic prostatitis/
	73	exp chronic pulmonary aspergillosis/
	74	exp multiple chronic conditions/
	75	exp chronic viral hepatitis/
	76	exp chronic vein insufficiency/
	77	exp chronic urticaria/
	78	exp chronic traumatic encephalopathy/
	79	exp chronic thromboembolic pulmonary hypertension/
	80	exp chronic stress/

	81	exp chronic sinusitis/
	82	exp chronic rhinosinusitis/
	83	exp chronic respiratory tract disease/
	84	exp chronic respiratory failure/
Intervention (Online support group)	85	"online support group".ab,ti.
	86	"online peer support".ab,ti.
	87	"online social support".ab,ti.
	88	(virtual adj3 group*).ab,ti.
	89	(virtual adj3 communit*).ab,ti.
	90	(virtual adj3 support*).ab,ti.
	91	(online adj3 group*).ab,ti.
	92	(online adj3 communit*).ab,ti.
	93	(online adj3 support*).ab,ti.
	94	(web adj4 group*).ab,ti.
	95	(internet adj4 communit*).ab,ti.
	96	(internet adj4 support*).ab,ti.
	97	(computer mediated adj3 group*).ab,ti.
	98	(computer mediated adj3 communit*).ab,ti.
	99	(computer mediated adj3 support*).ab,ti.
	100	(social media adj3 group*).ab,ti.
	101	(social media adj3 support*).ab,ti.
	102	(Facebook adj3 support*).ab,ti.
	103	"discussion forum*".ab,ti.
	104	"discussion fora".ab,ti.
	105	"bulletin board*".ab,ti.
	106	"Reddit".ab,ti.
	107	"chat room".ab,ti.
	108	"message board*".ab,ti.

	109	exp online support group/
Population Combination	110	1 or 2 or 3 or 4 or 5 or 6 or 7 or 8 or 9 or 10 or 11 or 12 or 13 or 14 or 15 or 16 or 17 or 18 or 19 or 20 or 21 or 22 or 23 or 24 or 25 or 26 or 27 or 28 or 29 or 30 or 31 or 32 or 33 or 34 or 35 or 36 or 37 or 38 or 39 or 40 or 41 or 42 or 43 or 44 or 45 or 46 or 47 or 48 or 49 or 50 or 51 or 52 or 53 or 54 or 55 or 56 or 57 or 58 or 59 or 60 or 61 or 62 or 63 or 64 or 65 or 66 or 67 or 68 or 69 or 70 or 71 or 72 or 73 or 74 or 75 or 76 or 77 or 78 or 79 or 80 or 81 or 82 or 83 or 84
Intervention Combination	110	85 or 86 or 87 or 88 or 89 or 90 or 91 or 92 or 93 or 94 or 94 or 96 or 97 or 98 or 99 or 100 or 101 or 102 or 103 or 104 or 105 or 106 or 107 or 108 or 109
Population AND Intervention Combination	111	109 and 110

Table 3: Search terms for Medline

Ovid MEDLINE® ALL 1946 to September 10, 2024		
**Concept**	**Search Number**	**Query**
Population (Individuals with a Chronic Condition)	1	(chronic adj illness*).ab,ti.
	2	(chronic adj disease*).ab,ti.
	3	(chronic adj condition*).ab,ti.
	4	(chronic adj infection*).ab,ti.
	5	(chronic adj health*).ab,ti.
	6	(chronic adj respiratory*).ab,ti.
	7	(chronic adj neurologic*).ab,ti.
	8	(chronic adj pain*).ab,ti.
	9	(chronic adj fatigue*).ab,ti.
	10	(long term adj2 condition*).ab,ti.
	11	"diabetes".ab,ti.
	12	"hypertension".ab,ti.
	13	"angina".ab,ti.
	14	"cardiovascular disease".ab,ti.
	15	"chronic obstructive pulmonary disease".ab,ti.
	16	"multiple sclerosis".ab,ti.
	17	"arthritis".ab,ti.
	18	"cancer".ab,ti.
	19	"irritable bowel syndrom*".ab,ti.
	20	"myalgic encephalomyelitis".ab,ti.
	21	"CFS".ab,ti.
	22	"ME/CFS".ab,ti.
	23	"long covid".ab,ti.

24	"coronary heart disease".ab,ti.
25	"cystic fibrosis".ab,ti.
26	"ulcerative colitis".ab,ti.
27	"chronic kidney disease".ab,ti.
28	"haemophilia".ab,ti.
29	"lupus".ab,ti.
30	"HIV".ab,ti.
31	"Crohn's disease".ab,ti.
32	"sickle cell anemia".ab,ti.
33	"fibromyalgia".ab,ti.
34	"inflammatory bowel disease".ab,ti.
35	"systemic sclerosis".ab,ti.
36	exp Chronic Disease/
37	exp "Chronic Kidney Disease-Mineral and Bone Disorder"/
38	exp Chronic Limb-Threatening Ischemia/
39	exp Chronic Pain/
40	exp Chronic Periodontitis/
41	exp Chronic Traumatic Encephalopathy/
42	exp Chronic Urticaria/
43	exp Fatigue Syndrome, Chronic/
44	exp Granulomatous Disease, Chronic/
45	exp Hepatitis B, Chronic/
46	exp Hepatitis C, Chronic/
47	exp Hepatitis, Chronic/
48	exp Hepatitis D, Chronic/
49	exp Kidney Failure, Chronic/
50	exp Leukemia, Lymphocytic, Chronic, B-Cell/
51	exp Leukemia, Myelogenous, Chronic, BCR-ABL Positive/

	52	exp Leukemia, Myeloid, Chronic-Phase/
	53	exp Leukemia, Myelomonocytic, Chronic/
	54	exp Multiple Chronic Conditions/
	55	exp Multiple Sclerosis, Chronic Progressive/
	56	exp Renal Insufficiency, Chronic/
	57	exp Pancreatitis, Chronic/
	58	exp Polyradiculoneuropathy, Chronic Inflammatory Demyelinating/
	59	exp Pulmonary Disease, Chronic Obstructive/
Intervention (Online support group)	60	"online support group".ab,ti.
	61	"online peer support".ab,ti.
	62	"online social support".ab,ti.
	63	(virtual adj3 group*).ab,ti.
	64	(virtual adj3 communit*).ab,ti.
	65	(virtual adj3 support*).ab,ti.
	66	(online adj3 group*).ab,ti.
	67	(online adj3 communit*).ab,ti.
	68	(online adj3 support*).ab,ti.
	69	(web adj4 group*).ab,ti.
	70	(web adj4 communit*).ab,ti.
	71	(internet adj4 communit*).ab,ti.
	72	(internet adj4 support*).ab,ti.
	73	(computer mediated adj3 group*).ab,ti.
	74	(computer mediated adj3 communit*).ab,ti.
	75	(computer mediated adj3 support*).ab,ti.
	76	(social media adj3 group*).ab,ti.
	77	(social media adj3 support*).ab,ti.
	78	(Facebook adj3 support*).ab,ti.
	79	"discussion forum*".ab,ti.

	80	"discussion fora".ab,ti.
	81	"bulletin board*".ab,ti.
	82	"Reddit".ab,ti.
	83	"chat room".ab,ti.
	84	"message board*".ab,ti.
Population Combination	85	1 or 2 or 3 or 4 or 5 or 6 or 7 or 8 or 9 or 10 or 11 or 12 or 13 or 14 or 15 or 16 or 17 or 18 or 19 or 20 or 21 or 22 or 23 or 24 or 25 or 26 or 27 or 28 or 29 or 30 or 31 or 32 or 33 or 34 or 35 or 36 or 27 or 38 or 39 or 40 or 41 or 42 or 43 or 44 or 45 or 46 or 47 or 48 or 49 or 50 or 51 or 52 or 53 or 54 or 55 or 56 or 57 or 58 or 59
Intervention Combination	86	60 or 61 or 62 or 63 or 64 or 65 or 66 or 67 or 68 or 69 or 70 or 71 or 72 or 73 or 74 or 75 or 76 or 77 or 78 or 79 or 80 or 81 or 82 or 83 or 84
Population AND Intervention Combination	87	85 and 86

Table 4: PsycInfo search terms

APA PsycInfo 1806 to September Week 1 2024		
**Concept**	**Search Number**	**Query**
Population (Individuals with a Chronic Condition)	1	(chronic adj illness*).ab,ti.
	2	(chronic adj disease*).ab,ti.
	3	(chronic adj condition*).ab,ti.
	4	(chronic adj infection*).ab,ti.
	5	(chronic adj health*).ab,ti.
	6	(chronic adj respiratory*).ab,ti.
	7	(chronic adj neurologic*).ab,ti.
	8	(chronic adj pain*).ab,ti.
	9	(chronic adj fatigue*).ab,ti.
	10	(long term adj2 condition*).ab,ti.
	11	"diabetes".ab,ti.
	12	"hypertension".ab,ti.
	13	"angina".ab,ti.
	14	"cardiovascular disease".ab,ti.
	15	"chronic obstructive pulmonary disease".ab,ti.
	16	"multiple sclerosis".ab,ti.
	17	"arthritis".ab,ti.
	18	"cancer".ab,ti.
	19	"irritable bowel syndrom*".ab,ti.
	20	"myalgic encephalomyelitis".ab,ti.
	21	"CFS".ab,ti.
	22	"ME/CFS".ab,ti.
	23	"long covid".ab,ti.

	24	"coronary heart disease".ab,ti.
	25	"cystic fibrosis".ab,ti.
	26	"ulcerative colitis".ab,ti.
	27	"chronic kidney disease".ab,ti.
	28	"haemophilia".ab,ti.
	29	"lupus".ab,ti.
	30	"HIV".ab,ti.
	31	"Crohn's disease".ab,ti.
	32	"sickle cell anemia".ab,ti.
	33	"fibromyalgia".ab,ti.
	34	"inflammatory bowel disease".ab,ti.
	35	"systemic sclerosis".ab,ti.
	36	exp Chronic Fatigue Syndrome/
	37	exp Chronic Illness/
	38	exp Chronic Mental Illness/
	30	exp Chronic Obstructive Pulmonary Disease/
	40	exp Chronic Pain/
	41	exp Chronic Stress/
	42	"online support group".ab,ti.
Intervention (Online support group)	43	"online peer support".ab,ti.
	44	"online social support".ab,ti.
	45	(virtual adj3 group*).ab,ti.
	46	(virtual adj3 communit*).ab,ti.
	47	(virtual adj3 support*).ab,ti.
	48	(online adj3 group*).ab,ti.
	49	(online adj3 communit*).ab,ti.
	50	(online adj3 support*).ab,ti.
	51	(web adj4 group*).ab,ti.

	52	(internet adj4 group*).ab,ti.
	53	(internet adj4 communit*).ab,ti.
	54	(internet adj4 support*).ab,ti.
	55	(computer mediated adj3 group*).ab,ti.
	56	(computer mediated adj3 communit*).ab,ti.
	57	(computer mediated adj3 support*).ab,ti.
	58	(social media adj3 group*).ab,ti.
	59	(social media adj3 support*).ab,ti.
	60	(Facebook adj3 support*).ab,ti.
	61	"discussion forum*".ab,ti.
	62	"discussion fora".ab,ti.
	63	"bulletin board*".ab,ti.
	64	"Reddit".ab,ti.
	65	"chat room".ab,ti.
	66	"message board*".ab,ti.
	67	exp Online Social Networks/
	68	exp Online Community/
	69	exp Computer Mediated Communication/
Population Combination	70	1 or 2 or 3 or 4 or 5 or 6 or 7 or 8 or 9 or 10 or 11 or 12 or 13 or 14 or 15 or 16 or 17 or 18 or 19 or 20 or 21 or 22 or 23 or 24 or 25 or 26 or 27 or 28 or 29 or 30 or 31 or 32 or 33 or 34 or 35 or 36 or 37 or 38 or 39 or 40 or 41
Intervention Combination	71	43 or 44 or 45 or 46 or 47 or 48 or 49 or 50 or 51 or 52 or 53 or 54 or 55 or 56 or 57 or 58 or 59 or 60 or 61 or 62 or 63 or 64 or 65 or 65 or 66 or 67 or 68 or 69
Population AND Intervention Combination	72	70 and 71

Table 5: Web of Science search terms

Web of Science Core Collection		
**Concept**	**Search Query**	**Query**
Population (Individuals with a Chronic Condition)	1	(chronic NEAR/1 illness*)
	2	(chronic NEAR/1 disease*)
	3	(chronic NEAR/1 condition*)
	4	(chronic NEAR/1 infection*)
	5	(chronic NEAR/1 health*)
	6	(chronic NEAR/1 respiratory*)
	7	(chronic NEAR/1 neurologic*)
	8	(chronic NEAR/1 pain*)
	9	(chronic NEAR/1 fatigue*)
	10	(long term NEAR/2condition*)
	11	"diabetes"
	12	"hypertension"
	13	"angina"
	14	"cardiovascular disease"
	15	"chronic obstructive pulmonary disease"
	16	"multiple sclerosis"
	17	"arthritis"
	18	"cancer"
	19	"irritable bowel syndrom*"
	20	"myalgic encephalomyelitis"
	21	"CFS"
	22	"ME/CFS"
	23	"long covid"

	24	"coronary heart disease"
	25	"cystic fibrosis"
	26	"ulcerative colitis"
	27	"chronic kidney disease"
	28	"haemophilia"
	29	"lupus"
	30	"HIV"
	31	"Crohn's disease"
	32	"sickle cell anemia"
	33	"fibromyalgia"
	34	"inflammatory bowel disease"
	35	"systemic sclerosis"
Intervention (Online support group)	36	"online support group"
	37	"online peer support"
	38	"online social support"
	39	(virtual NEAR/3 group*)
	40	(virtual NEAR/3 communit*)
	41	(virtual NEAR/3 support*)
	42	(online NEAR/3 group*)
	43	(online NEAR/3 communit*)
	44	(online NEAR/3 support*)
	45	(web NEAR/4 group*)
	46	(web NEAR/4 communit*)
	47	(web NEAR/4 support*)
	48	(internet NEAR/4 group*)
	49	(internet NEAR/4 communit*)
	50	(internet NEAR/4 support*)
	51	(computer mediated NEAR/4 group*)

	52	(computer mediated NEAR/4 communit*)
	53	(computer mediated NEAR/4 support*)
	54	(social media NEAR/3 group*)
	55	(social media NEAR/3 support*)
	56	(Facebook NEAR/3 support*)
Population Combination	57	"discussion forum*"
	58	“discussion fora”
	59	"bulletin board*"
	60	"Reddit"
	61	"chat room".
	62	"message board*"
	63	1 or 2 or 3 or 4 or 5 or 6 or 7 or 8 or 9 or 10 or 11 or 12 or 13 or 14 or 15 or 16 or 17 or 18 or 19 or 20 or 21 or 22 or 23 or 24 or 25 or 26 or 27 or 28 or 29 or 30 or 31 or 32 or 33 or 34 or 35
Intervention Combination	64	36 or 37 or 38 or 39 or 40 or 41 or 42 or 43 or 44 or 45 or 46 or 47 or 48 or 49 or 50 or 51 or 52 or 53 or 54 or 55 or 56 or 57 or 58 or 59 or 60 or 61 or 62
Population AND Intervention Combination	65	63 and 64

Table 6: Grey Literature; Pre-Publication Databases and Google Scholar Searches

**Search Type**	**Search Engine**	Search Terms
Articles	Google Scholar (September 2024)	("chronic illness" OR "chronic disease" OR "chronic condition" OR "chronic pain") AND ("online support group" OR "online peer support")
Pre-Publications	MedRxiv (September 2024)	("chronic illness" OR "chronic disease" OR "chronic condition") AND ("online support group" OR "online peer support")
	PsyArXiv (September 2024)	Chronic condition AND online support group
Grey Literature	Google Advanced Search (September 2024)	("chronic illness" OR "chronic disease" OR "chronic condition" OR "chronic pain") AND ("online support group" OR "online peer support")
	EThOS (February 2023)	Chronic condition AND online support group
		Chronic illness AND online support group
		Chronic disease AND online support group
		Chronic pain AND online support group
		Chronic condition AND online peer support

		Chronic illness AND online peer support
		Chronic disease AND online peer support
		Chronic pain AND online peer support

Table 7: Reasons for exclusion in full text screening

Reason for exclusion	Papers excluded
Wrong publication (e.g., meeting or conference abstract, review)	**[1-72]**
Wrong intervention (e.g., not measuring online support group; blog; online therapy; social media)	**[73-125]**
Wrong population (e.g., caregivers, survivors, prior to becoming ill)	**[126-135]**
Wrong outcome (e.g., not health outcome or not looking at effect of online support group on health outcome)	**[136-160]**
Wrong method (e.g., content analysis)	**[161-173]**
Complex intervention	**[174-203]**
Foreign language	**[204-207]**
Duplicate of included / excluded study (e.g., PhD thesis of published paper or published paper of PhD)	**[208-212]**
Can’t find/access	**[213-229]**

References:

1. Basile, M., et al., *Providing social support for adults with cystic fibrosis during the COVID-19 pandemic: Pilot study of a web-based support group.* American Journal of Respiratory and Critical Care Medicine. Conference: American Thoracic Society International Conference, ATS, 2021. **203**(9).
2. Geller, H.M., et al., *Evaluating the contribution of virtual peer-led support to comprehensive prostate cancer (PCa) care: The AnCan experience.* Journal of Clinical Oncology. Conference, 2022. **40**(6).
3. Harkin, L., et al., *Experiencing online cancer communities-a qualitative study.* European Journal of Cancer, 2015. **3**: p. S134-S135.
4. Henthorne, K., et al., *75 Accesstoeducation and support for adults with cystic fibrosis byvirtual support group with a focus on readiness for lung transplant.* Journal of Cystic Fibrosis, 2021. **20**: p. S37-S38.
5. Huber, J., et al., *A cross-sectional comparison study of self-help for prostate cancer: How do online and face-to-face support groups differ?* Journal of Urology, 2015. **1**: p. e308.
6. Huber, J., et al., *Online vs. Face-to-face support groups for prostate cancer: A cross-sectional comparison study.* European Urology, Supplements, 2015. **14**: p. e374.
7. Hunter-Smith, A., et al., *Examining social media peer support and improving psychosocial outcomes for young women with breast cancer.* Journal of Clinical Oncology. Conference, 2021. **39**(28).
8. Khan, Y., et al., *Participation in a virtual community and its impact on quality of life and perceived support.* Circulation: Cardiovascular Quality and Outcomes. Conference: American Heart Association's Quality of Care and Outcomes Research, 2017. **10**.
9. Kyriakides, S., *The role of cancer patient support groups.* European Journal of Cancer, 2014. **2**: p. S43-S44.
10. Lepore, S.J., et al., *Preliminary findings from a randomized trial of standard versus prosocial online support groups for distressed breast cancer survivors.* Asia-Pacific Journal of Clinical Oncology, 2012. **3**: p. 167.
11. Martin, M.L., et al., *Evaluating the user-perceived benefit of a virtual lung cancer patient education and support community: LVNG With Lung Cancer.* Journal of Clinical Oncology. Conference: Annual Meeting of the American Society of Clinical Oncology, ASCO, 2021. **39**(15).
12. Mehring, S., *Connecting on social networks improves patient and caregiver quality of life.* Pediatric Blood and Cancer, 2011. **57**: p. 851.
13. Montague, D., *The ALK Positive UK charity and Support Group: the health and well-being benefits for patients and their families.* Lung Cancer, 2020. **139**: p. S74.
14. Oser, T.K., et al., *Social Media in the Diabetes Community: a Novel Way to Assess Psychosocial Needs in People with Diabetes and Their Caregivers.* Current Diabetes Reports, 2020. **20**.
15. Perkins, R., et al., *Five clicks apart: Reported use of social media among adolescents and young adults with CF.* Pediatric Pulmonology, 2019. **54**: p. 398-399.
16. Redway, A., et al., *MA08.09 The Role of Social Media as a Platform for Patient-Led Support Groups.* Journal of Thoracic Oncology, 2022. **17**: p. S74.
17. Savin, K., *Empowering young women with type 1 diabetes: Evaluating a peer-led diabetes support group.* Diabetes, 2013. **1**: p. A637.
18. Seckin, G., *Finding meanings in coping with cancer in old age and self-reported benefits of participation in online cancer support groups.* Psycho-Oncology, 2010. **2**: p. S124-S125.
19. Seckin, G., *Does it matter whether it is online or offline: Testing relative significance of alternative sources of supportive relations in cancer care of aging adults.* Supportive Care in Cancer, 2013. **1**: p. S290-S291.

20. Von Hippel, C.D., et al., *A qualitative exploration of self-developed and peer-recommended techniques used by women with breast cancer to improve sexual functioning during and after treatment.* Cancer Research. Conference, 2018. **79**(4).
21. Huntley, A., *Prostate cancer: Online support reduces distress in men with prostate cancer.* Nature Reviews Urology, 2016. **13**(1): p. 9-10.
22. Cohen, J., P. Patterson, and O. Husson, *Canteen Connect: The evaluation of an online health community for AYAs impacted by cancer.* Asia-Pacific Journal of Clinical Oncology, 2021. **17**: p. 12-12.
23. Smit, W.M., et al., *Patients with breast cancer feel empowered by their participation in online support groups.* Journal of Clinical Oncology, 2007. **25**(18): p. 1.
24. Anzaldi, L., A. Adams-Clark, and A. Kaplin, *Helping MS patients find their purpose in life: A pilot study of the effect of participation in an online community.* Annals of Neurology, 2016. **80**: p. S117.
25. Barnard, M.L., et al., *Online support for continuing learning: A Moodle resource for expert patients with Type 1 diabetes.* Diabetic Medicine, 2009. **1**: p. 169-170.
26. Brown, E.K., et al., *A novel support group model for patients with Lynch syndrome.* Familial Cancer, 2011. **10**: p. 722.
27. Welten, V.M., et al., *Patient Empowerment on the Rectal Cancer Treatment Journey: A Call for Advocacy and Peer Support.* Annals of Surgical Oncology, 2022. **29**: p. S431-S432.
28. Keeling, D., et al., *Function, Content and Process in Online Health Communities: Implications for e-Health*. 2008, Ios Press: Amsterdam. p. 5-13.
29. Abbott, J. and K. Beattie, *124 Engaging medical teams in support group recommendations.* Lung Cancer, 2021. **156**: p. S51-S52.
30. Almanea, A., P. Bath, and L. Sbaffi, *Empowering people with type two diabetes through online support groups.* Computer Methods and Programs in Biomedicine, 2019. **171**: p. 3.
31. An, L., et al., *An exploratory survey of the construction of peer support based on smart phones.* Diabetes/Metabolism Research and Reviews, 2015. **31**: p. 13-14.
32. Anonymous, *International models of collaboration: Research, training and practice in professionally-led online support groups.* Psycho-Oncology, 2010. **2**: p. S37.
33. Attai, D.J., et al., *#BCSM: Using social media to develop a novel breast cancer support community.* Annals of Surgical Oncology, 2014. **2**: p. 24.
34. Beattie, N.M. and L. Lloyd, *Rising phoenix suport group.* Asia-Pacific Journal of Clinical Oncology, 2009. **2**: p. A205-A206.
35. Blackstock, S., et al., *The use of a WhatsAppTM broadcast group to improve knowledge and engagement of adolescents with type 1 diabetes.* Archives of Disease in Childhood, 2016. **101**: p. A315-A316.
36. Bortz, A., C. Frail, and K. Funk, *Use of a social media platform for a pharmacist-led diabetes support group: Lessons learned.* Journal of the American Pharmacists Association. Conference, 2017. **57**(3).
37. Broussard, S.B., et al., *Virtual psychosocial services for the adult population in a large community oncology practice.* Journal of Clinical Oncology. Conference, 2021. **39**(28).
38. Butow, P., et al., *Cancer support group research: An example of collaborative work in Australia.* Psycho-Oncology, 2009. **2**: p. S30.
39. Chan, H.Y., et al., *Effects of a structured web-based support group for adolescents with cancer: A pilot study.* Pediatric Blood and Cancer, 2013. **3**: p. 226-227.
40. Cheng, K.Y., Y. Chen, and C.F. Chen, *From "being Helped" to "mutual Help": Effectiveness evaluation on self-help group.* Journal of Global Oncology, 2018. **4**: p. 104s.
41. Cheshire, J., et al., *The development of an online psychological support intervention for teenagers and young adults (TYA).* Pediatric Blood and Cancer, 2014. **2**: p. S152.

42. Classen, C., et al., *GyneGals: An online support group for women who are sexually distressed following treatment for gynecologic cancer.* Psycho-Oncology, 2016. **25**: p. 132.
43. Classen, C., et al., *The development and pilot testing of a web-based support group for women with sexual problems due to gynecologic cancer.* Psycho-Oncology, 2010. **2**: p. S109.
44. Cockle-Hearne, J., et al., *Online self-management for distress after prostate cancer treatment: Assessing acceptability and viability of The Getting Down to Coping Programme.* Psycho-Oncology, 2016. **25**: p. 110.
45. Cusimano, M.C., et al., *Association of treatment modality with sexual dysfunction in gynecologic cancer survivors: A secondary analysis of the gyne-GALS randomized controlled trial.* Gynecologic Oncology, 2020. **159**: p. 58.
46. Des Bordes, J., et al., *Physical challenges in RA: A qualitative study of an online patient support group.* Arthritis and Rheumatology, 2019. **71**: p. 3972-3973.
47. McNamara, N., *Online support groups for young people with eating disorders: Benefits and challenges.* European Child and Adolescent Psychiatry, 2013. **1**: p. S168.
48. Northern, A., et al., *How do patient attendees rate virtual group education: Our initial findings?* Diabetic Medicine, 2021. **38**: p. 67.
49. Petrovski, G., *Use of social media for improving glucose control in patients with type 1 diabetesuse of social media for improving glucose control in patients with type 1 diabetes.* Hormone Research in Paediatrics, 2018. **90**: p. 24.
50. Petrovski, G., *Education through facebook and other social media.* Diabetes Technology and Therapeutics, 2019. **21**: p. A9.
51. Stephen, J., et al., *Benefits of participation in professionally-facilitated online support groups: Quantitative and qualitative outcomes for patients, survivors and family caregivers.* Asia-Pacific Journal of Clinical Oncology, 2012. **3**: p. 143.
52. Finfgeld, D.L., *Therapeutic groups online: The good, the bad, and the unknown.* Issues in Mental Health Nursing, 2000. **21**(3): p. 241-255.
53. ro, L., *Alone together. Cancer patients and survivors find treatment--and support--online. It can make all the difference.* 1999. **1**(1): p. 59-63.
54. Chang, P.F. and N.N. Bazarova, *CHRONIC PAIN COMMUNICATION AND SOCIAL SUPPORT IN ONLINE AND MOBILE INTERACTIONS.* Gerontologist, 2015. **55**: p. 511-511.
55. Hargreaves, S. and P.A. Bath, *Online health forums: the role of online support for people living with breast cancer.* Breast Cancer Management, 2019. **8**(2): p. 4.
56. Haun, M.W., et al., *The era of the digital natives is approaching: Insights into online peer-to-peer support for persons affected by prostate cancer.* World Journal of Urology, 2020. **38**(10): p. 2433-2434.
57. Huntley, A., *Online support reduces distress in men with prostate cancer.* Nature Reviews Urology, 2016. **13**(1): p. 9-U24.
58. Januzik, T. *Online support group for adolescents aged 10-14 with chronic illness*. 2020 2020.
59. Foster, C., et al., *Restore: Testing feasibility and acceptability of an online intervention to support self-management of cancer-related fatigue in a multi-centre proof of concept randomised controlled trial.* Psycho-Oncology, 2014. **3**: p. 262.
60. Rose, L., et al., *50524 Facebook groups provide support and product recommendations for patients using scalp cooling to prevent chemotherapy-induced alopecia: A survey study.* Journal of the American Academy of Dermatology, 2024. **91**(3): p. AB45.
61. Abioye, O., et al., *Cancer health disparities in minority communities: peer support networks can bridge the gap.* Cancer Causes Control, 2024. **35**(11): p. 1407-1411.
62. Wainwright, J., et al., *P400 “It’s good to talk”: the development and acceptability of online group support sessions for adults with CF in a large adult CF centre in the UK.* Journal of Cystic Fibrosis, 2024. **23**: p. S190.

63. Sinnott, S. and K. Smith, *P401 Making connections: the implementation of an age specific youth group across Southampton Children’s Hospital’s Cystic Fibrosis (CF) service.* Journal of Cystic Fibrosis, 2024. **23**: p. S191.
64. Houssem, A., et al., *P121â€…Online support group for lupus patients: the Tunisian experience.* Lupus Science & Medicine, 2024. **11**(Suppl 1): p. null.
65. Harrison, V., et al., *136 Supporting the wellbeing of oncodriven lung cancer patients online: a qualitative study.* Lung Cancer, 2024. **190**: p. 107697.
66. Wendy, H.S.H., *An evaluation study on the distress level and perceived outcomes of head and neck cancer patient support group.* Journal of Medical Imaging and Radiation Sciences, 2023. **54**(2, Supplement 1): p. S6.
67. Davies, E., *Ambivalent Speculations: Learning to Live with Barrett's Esophagus in the UK Using Facebook Support Groups.* Medical Anthropology: p. 1-14.
68. Schneider, B., et al., *POS0064-PARE PATIENT REPORTED BENEFITS OF PARTICIPATING IN AN ONLINE HEALTH COMMUNITY IN MANAGING RHEUMATOID ARTHRITIS.* Annals of the Rheumatic Diseases, 2023. **82**(Suppl 1): p. 243-243.
69. Reifegerste, D., K. Wendt, and P. Stehr, *Between cat content and Yeswecan!cer: Digital media repertoires for disease management of young cancer patients.* Studies in Communication and Media, 2023. **12**: p. 236-241.
70. Samalin, E., et al., *P-102 Unmet needs of patients during colorectal cancer care and treatment – a qualitative study through an online patient community.* Annals of Oncology, 2023. **34**: p. S50.
71. Reidy, C., et al., *Peer support in chronic health conditions.* BMJ, 2024. **386**: p. e070443.
72. Lehardy, E.N. and B.J. Fowers, *Ultimate (evolutionary) explanations for the attraction and benefits of chronic illness support groups: Attachment, belonging, and collective identity.* Current Psychology, 2020. **39**(4): p. 1405-1415.
73. Kostlin, D., B. Siem, and A. Rohmann, *Social support in online peer groups for celiac disease.* European Journal of Health Psychology, 2023: p. No Pagination Specified.
74. Zhu, Y., E.M. Glowacki, and Y. Yang, *A social ties-based approach to breast cancer patients' quality of life: Examining group ties and individual ties across offline and online settings.* Health Communication, 2021. **36**(6): p. 741-751.
75. Malik, F.S., et al., *Augmenting Traditional Support Groups for Adolescents With Type 1 Diabetes Using Instagram: Mixed Methods Feasibility Study.* JMIR Diabetes, 2021. **6**(4): p. e21405.
76. Han, X., et al., *Weibo friends with benefits for people live with HIV/AIDS? The implications of Weibo use for enacted social support, perceived social support and health outcomes.* Social Science and Medicine, 2018. **211**: p. 157-163.
77. Hurley-Wallace, A., S. Kirby, and F. Bishop, *Trusting in the online 'community': An interview study exploring internet use in young people with chronic pain.* British Journal of Pain, 2022. **16**: p. 341-353.
78. Kim, J.N. and S. Lee, *Communication and cybercoping: coping with chronic illness through communicative action in online support networks.* Journal of health communication, 2014. **19**: p. 775-794.
79. Lazard, A.J., et al., *Using social media for peer-to-peer cancer support: Interviews with young adults with cancer.* JMIR Cancer, 2021. **7**.
80. Palant, A. and W. Himmel, *Are there also negative effects of social support? A qualitative study of patients with inflammatory bowel disease.* BMJ Open, 2019. **9**.
81. Broom, A., *Virtually He@lthy: The Impact of Internet Use on Disease Experience and the Doctor-Patient Relationship.* Qualitative Health Research, 2005. **15**(3): p. 325-345.
82. Kavathe, R., *Patterns of access and use of online health information among Internet users: A case study.* Dissertation Abstracts International Section A: Humanities and Social Sciences, 2010. **70**(9): p. 3278.

83. Audrain-Pontevia, A.F., L. Menvielle, and M. Ertz, *Effects of Three Antecedents of Patient Compliance for Users of Peer-to-Peer Online Health Communities: Cross-Sectional Study.* Journal of Medical Internet Research, 2019. **21**(11): p. e14006.
84. Mulvaney, S.A., et al., *An Internet-Based Program to Improve Self-Management in Adolescents With Type 1 Diabetes.* Diabetes Care, 2010. **33**(3): p. 602-604.
85. Witt, D., et al., *Measures of Patient Activation and Social Support in a Peer-Led Support Network for Women With Cardiovascular Disease.* Journal of Cardiopulmonary Rehabilitation and Prevention, 2016. **36**(6): p. 430-437.
86. Hansen, D.L., *Knowledge sharing, maintenance, and use in online support communities.* Dissertation Abstracts International Section A: Humanities and Social Sciences, 2008. **68**(10): p. 4118.
87. Seckin, G., *Using Hierarchical Multivariate Analysis to Examine Interactions Between Alternative Appraisals of Cancer and Virtual Health Support Among Middle-Aged and Older Internet Users: Predicting Positive Self-Reappraisal After Cancer.* Research on Aging, 2013. **35**(6): p. 688-709.
88. Arnold, S., et al., *Living with vulval lichen sclerosus: a qualitative interview study*.* British Journal of Dermatology, 2022. **187**: p. 909-918.
89. Gardner, T., et al., *The Effect of Adjunct Telephone Support on Adherence and Outcomes of the Reboot Online Pain Management Program: Randomized Controlled Trial.* Journal of Medical Internet Research, 2022. **24**.
90. Kalichman, S.C., et al., *Health-related internet use, coping, social support, and health indicators in people living with HIV/AIDS: Preliminary results from a community survey.* Health Psychology, 2003. **22**: p. 111-116.
91. Nguyen, H.Q., et al., *Is Internet-based support for dyspnea self-management in patients with chronic obstructive pulmonary disease possible? Results of a pilot study.* Heart and Lung: Journal of Acute and Critical Care, 2005. **34**: p. 51-62.
92. Owen, J.E., et al., *Improving the effectiveness of adjuvant psychological treatment for women with breast cancer: The feasibility of providing online support.* Psycho-Oncology, 2004. **13**: p. 281-292.
93. Allen, C., et al., *The contribution of internet use in personal networks of support for long-term condition self-management.* Chronic Illness, 2019. **15**(3): p. 220-235.
94. Baldwin, P.A., et al., *A web-based mental health intervention to improve social and occupational functioning in adults with type 2 diabetes (The Springboard Trial): 12-month outcomes of a randomized controlled trial.* Journal of Medical Internet Research Vol 22(12), 2020, ArtID e16729, 2020. **22**(12).
95. Ure, C., *Living with and beyond breast cancer: Exploring women's use of social media to support psychosocial health.* Dissertation Abstracts International: Section B: The Sciences and Engineering, 2022. **83**(6): p. No Pagination Specified.
96. Zhu, Y., et al., *Relationships among patient activation, social support and online health information seeking of community-dwelling older adults living with coronary heart disease.* Journal of Advanced Nursing, 2023. **79**(1): p. 161-169.
97. Ure, C., et al., *Exploring Strategies for Using Social Media to Self-Manage Health Care When Living With and Beyond Breast Cancer: In-Depth Qualitative Study.* Journal of Medical Internet Research, 2020. **22**(5): p. e16902.
98. Muir, J., et al., *Exploring the role of online health information and social media in the illness experience of arthritis-related fatigue: A focus group study.* Musculoskeletal Care, 2020. **18**(4): p. 501-509.
99. Fogel, J., et al., *Racial/ethnic differences and potential psychological benefits in use of the internet by women with breast cancer.* 2003. **1**(2): p. 107-17.
100. Wright, K.B. and S.A. Rains, *Weak-Tie Support Network Preference, Health-Related Stigma, and Health Outcomes in Computer-Mediated Support Groups.* Journal of Applied Communication Research, 2013. **41**(3): p. 309-324.
101. Hersh, A.C., *Social support online: Testing the effects of highly person-centered messages in breast cancer support groups.* Dissertation Abstracts International Section A: Humanities and Social Sciences, 2012. **72**(9): p. 3057.

102. Wu, J.J., et al., *Does Online Community Participation Contribute to Medication Adherence? An Empirical Study of Patients with Chronic Diseases.* Int J Environ Res Public Health, 2021. **18**(10).
103. Coppini, V., et al., *Patients' perspectives on cancer care disparities in Central and Eastern European countries: experiencing taboos, misinformation and barriers in the healthcare system.* Front Oncol, 2024. **14**: p. 1420178.
104. Chausset, A., et al., *Diagnosis journey for children with juvenile idiopathic arthritis: a qualitative study.* Arch Dis Child, 2024. **109**(12): p. 1003-1009.
105. Ding, Y., et al., *The relationship between perceived social support, coping style, and the quality of life and psychological state of lung cancer patients.* BMC Psychology, 2024. **12**(1): p. 439.
106. Gunen, B., et al., *Social Support and Dietary Behaviors Among Adults with ESKD and Obesity: TH-PO912.* Journal of the American Society of Nephrology, 2023. **34**: p. 346-347.
107. Lange-Drenth, L., et al., *Association of the Extent of Internet Use by Patients With Cancer With Social Support Among Patients and Change in Patient-Reported Treatment Outcomes During Inpatient Rehabilitation: Cross-sectional and Longitudinal Study.* JMIR Cancer, 2023. **9**: p. e39246.
108. Loeb, S., et al., *Qualitative Study on Internet Use and Care Impact for Black Men With Prostate Cancer.* Health Educ Behav, 2024. **51**(3): p. 359-366.
109. Peng, M., et al., *Analysis of the influential factors of the emotional health of patients with cancer based on the structural equation model: the role of social media and emotional support.* Support Care Cancer, 2023. **31**(7): p. 417.
110. Gandamihardja, T.A., et al., *The Role of Social Media and Breast Cancer: How Does It Impact Patients?* Breast Care, 2023. **18**(3): p. 203-208.
111. Köstlin, D., B. Siem, and A. Rohmann, *Social support in online peer groups for celiac disease.* European Journal of Health Psychology, 2023. **30**(3): p. 138-143.
112. Ahn, J. and K.E. Lee, *Experiences of peer support activities and the need for a metaverse-based program in young women with breast cancer: A qualitative study.* Asia Pac J Oncol Nurs, 2023. **10**(7): p. 100253.
113. Chen, Y.T., et al., *Peer-Led Symptom Management Intervention to Enhance Resilience in People With Systemic Sclerosis: Mediation Analysis From a Randomized Clinical Trial.* Arthritis Care Res (Hoboken), 2024. **76**(9): p. 1278-1286.
114. Wang, Y., S. Bao, and Y. Chen, *How does social media use influence the mental health of pancreatic cancer patients: a chain mediating effect of online social support and psychological resilience.* Front Public Health, 2023. **11**: p. 1166776.
115. Guo, Y., et al., *The influence of online social support on health self-management among gay men living with HIV in China.* Journal of Community & Applied Social Psychology, 2024. **34**(5): p. e2870.
116. Saab, R.H., et al., *Social media use is associated with higher levels of anxiety and depression in patients with neuroendocrine carcinoma of the cervix: A NeCTuR study.* Gynecol Oncol, 2023. **177**: p. 95-102.
117. Sydora, B.C., et al., *Challenges in diagnosis and health care in polycystic ovary syndrome in Canada: a patient view to improve health care.* BMC Women's Health, 2023. **23**(1): p. 569.
118. Halverson, C.M.E., T.A. Doyle, and S. Vershaw, *Social media use by patients with hypermobile Ehlers-Danlos syndrome.* Mol Genet Genomic Med, 2024. **12**(6): p. e2467.
119. Rambod, M., et al., *The effect of virtual interactive nurse-led support group intervention on fatigue, shock anxiety, and acceptance of implantable cardioverter defibrillator patients: a randomized trial.* BMC Cardiovasc Disord, 2024. **24**(1): p. 40.
120. Cook, A., *All in the Same Boat: A Qualitative Investigation into the Relationship Between Peer-Led Chronic Pain Support Groups and Chronic Pain Management*, in *Community Psychology, Counseling and Family Therapy*. 2022, St. Cloud State University.

121. Farr, M., et al., *Experiences of Patient-Led Chronic Pain Peer Support Groups After Pain Management Programs: A Qualitative Study.* Pain Medicine, 2021. **22**(12): p. 2884-2895.
122. Kulandaivelu, Y. and S.A. Kohut, *Peer Support for Adolescents with Chronic Illness*, in *Peer Support in Medicine: A Quick Guide*, J.D. Avery, Editor. 2021, Springer International Publishing: Cham. p. 95-113.
123. Yustisia, I., et al., *The Transformation of Digital Technology: Its Impact on Human Communication*. 2023.
124. Kirk, S. and L.J. Milnes, *Peer Support for Young People with Chronic Conditions*, in *Self-Management of Young People with Chronic Conditions: A Strength-Based Approach for Empowerment and Support*, J.N.T. Sattoe, A. van Staa, and S.R. Hilberink, Editors. 2021, Springer International Publishing: Cham. p. 135-160.
125. Zhao, J., et al., *Rehab-Diary: Enhancing Recovery Identity with an Online Support Group for Middle Aged and Older Ovarian Cancer Patients.* Proc. ACM Hum.-Comput. Interact., 2024. **8**(MHCI): p. Article 273.
126. Stephen, J., et al., *Evaluation of cancerchatcanada: A program of online support for canadians affected by cancer.* Current Oncology, 2013. **20**: p. 39-47.
127. Wiljer, D., et al., *A qualitative study of an internet-based support group for women with sexual distress due to gynecologic cancer.* Journal of cancer education : the official journal of the American Association for Cancer Education, 2011. **26**: p. 451-458.
128. Haldar, S., et al., *Use and impact of an online community for hospital patients.* Journal of the American Medical Informatics Association : JAMIA., 2020. **27**.
129. Kozlowski, D., et al., *Dusted community: Piloting a virtual peer-to-peer support community for people with an asbestos-related diagnosis and their families.* Journal of Psychosocial Oncology, 2014. **32**: p. 463-475.
130. Lepore, S.J., et al., *Digital literacy linked to engagement and psychological benefits among breast cancer survivors in Internet-based peer support groups.* European Journal of Cancer Care, 2019. **28**(4): p. 1-8.
131. Huang, X.M. and J. Fan, *Understand the Impact of Technology Feature in Online Health Communities: Why the Representation of Information Matters.* International Journal of Human-Computer Interaction, 2023. **39**(4): p. 691-706.
132. Liu, J.F. and J.Y. Wang, *Users' Intention to Continue Using Online Mental Health Communities: Empowerment Theory Perspective.* International Journal of Environmental Research and Public Health, 2021. **18**(18): p. 17.
133. Stephen, J., et al., *Talking with text: Communication in therapist-led, live chat cancer support groups.* Social Science & Medicine, 2014. **104**: p. 178-186.
134. Gonzales, B.R., et al., *Salud Latina: feasibility of a synchronous online chat for latinos at risk for type 2 diabetes.* Inform Health Soc Care, 2023. **48**(1): p. 95-107.
135. Pham, S., et al., *'No matter what time of day': The value of joining Facebook groups supporting women's self-management of gestational diabetes mellitus.* Health Expect, 2024. **27**(3): p. e14082.
136. Litchman, M.L., E. Rothwell, and L.S. Edelman, *The diabetes online community: Older adults supporting self-care through peer health.* Patient Education & Counseling, 2018. **101**(3): p. 518-523.
137. Marziali, E., *E-health program for patients with chronic disease.* Telemedicine Journal & E-Health, 2009. **15**(2): p. 176-81.
138. Reynolds, M., S. Driver, and M. Bennett, *The social network - using social media to support individuals with traumatic brain injury participating in a pilot study weight-loss program.* Brain Injury, 2018. **32**(12): p. 1450-1454.
139. Ahola Kohut, S., et al., *The internet as a source of support for youth with chronic conditions: A qualitative study.* Child: Care, Health and Development, 2018. **44**(2): p. 212-220.

140. Beczkiewicz, A.T.E., R.L. Scharff, and B.B. Kowalcyk, *Facilitating Evaluation of Hemolytic Uremic Syndrome Long-Term Health Outcomes Through Social Media Support Groups.* Frontiers in public health, 2020. **8**: p. 544154.
141. Lazard, A.J., et al., *Initiation and Changes in Use of Social Media for Peer Support among Young Adult Cancer Patients and Survivors.* Psycho oncology., 2021. **24**.
142. Xu, Y., et al., *Modeling intention to participate in face-to-face and online lung cancer support groups.* Psycho-Oncology, 2014. **23**: p. 555-561.
143. Gilmore, H., *Case study of learning and instruction for members of an online reflex sympathetic dystrophy support group.* Dissertation Abstracts International Section A: Humanities and Social Sciences, 2016. **77**(5): p. No Pagination Specified.
144. Stewart Loane, S., D'Aless, and S. ro, *Empowered and knowledgeable health consumers: The impact of online support groups on the doctor-patient relationship.* Australasian Marketing Journal (AMJ), 2014. **22**(3): p. 238-245.
145. Kanter, E., J.L. Bevan, and S.M. Dorros, *The Use of Online Support Groups to Seek Information about Chronic Illness: Applying the Theory of Motivated Information Management.* Communication Quarterly, 2019. **67**(1): p. 100-121.
146. Liu, N., Y. Tong, and H.C. Chan, *Information Seeking in Online Healthcare Communities: The Dual Influence From Social Self and Personal Self.* Ieee Transactions on Engineering Management, 2017. **64**(4): p. 529-538.
147. Chorbev, I., M. Sotirovska, and D. Mihajlov, *Virtual communities for diabetes chronic disease healthcare.* International Journal of Telemedicine & Applications, 2011. **2011**: p. 721654.
148. Han, J.Y., et al., *Expressing positive emotions within online support groups by women with breast cancer.* Journal of Health Psychology, 2008. **13**(8): p. 1002-1007.
149. Bender, J.L., et al., *What is the role of online support from the perspective of facilitators of face-to-face support groups? A multi-method study of the use of breast cancer online communities.* Patient Education and Counseling, 2013. **93**: p. 472-479.
150. des Bordes, J.K.A., et al., *Interactions and perceptions of patients with rheumatoid arthritis participating in an online support group.* Clinical rheumatology., 2020. **31**.
151. Haller, J., et al., *Impact of Pathologist Involvement in Sarcoma and Rare Tumor Patient Support Groups on Facebook: A Survey of 542 Patients and Family Members.* Archives of pathology & laboratory medicine, 2018. **142**: p. 1113-1119.
152. Williamson, L.D. and K. Prins, *Uncertain and Anxiously Searching for Answers: The Roles of Negative HealthCare Experiences and Medical Mistrust in Intentions to Seek Information from Online Spaces.* Health Commun, 2024. **39**(6): p. 1082-1093.
153. Tankha, H., et al., *A mixed-methods investigation into the us versus them mentality in Facebook groups for chronic pain.* Health Psychol, 2023. **42**(7): p. 460-471.
154. Ardissone, A., I. Leonowicz-Bukała, and M. Struck-Peregończyk, *"Can Anyone Tell Me…". Online Health Communities in Diabetes Self-Management in Poland and Italy.* Health Communication: p. 1-8.
155. Nguyen, A.L., et al., *A community-academic partnered approach to designing a Virtual Village to address the needs of older adults living with HIV.* AIDS Care, 2024. **36**(11): p. 1626-1634.
156. Alhaboby, Z.A., et al., *Cybervictimization of Adults With Long-term Conditions: Cross-sectional Study.* J Med Internet Res, 2023. **25**: p. e39933.
157. Winters, C.A. and T. Sullivan, *Chronic illness experience of isolated rural women: Use of an online support group intervention*, in *Rural Nursing: Concepts, Theory, and Practice*, Charlene A. Winters, Editor. 2013, Springer Publishing Company: New York.
158. van der Eijk, M., et al., *Using Online Health Communities to Deliver Patient-Centered Care to People With Chronic Conditions.* J Med Internet Res, 2013. **15**(6): p. e115.
159. Moreton, C.H.E. and P. Saukko, *Letting go of self-transformation? lurkers' tactics of body acceptance in and against an online support group for polycystic ovary syndrome.* Feminist Media Studies: p. 1-16.

160. Haber, T., et al., *'It's especially good just to know that you're not the only one': a qualitative study exploring experiences with online peer support programmes for the Fragile X community.* Journal of Intellectual Disability Research, 2025. **69**(1): p. 30-43.
161. Berard, A.A. and A.P. Smith, *Post Your Journey: Instagram as a Support Community for People With Fibromyalgia.* Qualitative health research, 2019. **29**: p. 237-247.
162. Gaulin, N.L., *The experience of adolescents using online social networks to cope with their cancer: Exploring grouploop's discussion board and online support groups.* Dissertation Abstracts International: Section B: The Sciences and Engineering, 2006. **66**(11): p. 6270.
163. Nicholas, D.B., et al., *Evaluation of an online peer support network for adolescents with chronic kidney disease.* Journal of Technology in Human Services, 2009. **27**(1): p. 23-33.
164. Loane, S.S., d'Aless, and S. ro, *Peer-to-peer value through social capital in an online motor neuron disease community.* Journal of Nonprofit & Public Sector Marketing, 2013. **25**(2): p. 164-185.
165. van der Eijk, M., et al., *Using online health communities to deliver patient-centered care to people with chronic conditions.* Journal of medical Internet research, 2013. **15**: p. e115.
166. Yan, L. and Y. Tan, *Feeling Blue? Go Online: An Empirical Study of Social Support Among Patients.* Information Systems Research, 2014. **25**(4): p. 690-709.
167. Hoybye, M.T., C. Johansen, and T. Tjornhoj-Thomsen, *Online interaction. Effects of storytelling in an internet breast cancer support group.* Psycho-Oncology, 2005. **14**(3): p. 211-20.
168. Willis, E., *The making of expert patients: The role of online health communities in arthritis self-management.* Journal of Health Psychology, 2014. **19**(12): p. 1613-1625.
169. Bar-Lev, S., *"We are here to give you emotional support": Performing emotions in an online HIV/AIDS support group.* Qualitative Health Research, 2008. **18**: p. 509-521.
170. Murray, R. and L. Turner, *Using Communities of Practice Theory to Understand the Crisis of Identity in Chronic Fatigue Syndrome/Myalgic Encephalomyelitis (CFS/ME).* Chronic Illness, 2023. **19**(1): p. 56-64.
171. Lindgreen, P., et al., *Discussions About Binge Eating and Type 2 Diabetes in a Facebook Group: A Qualitative Analysis.* Int J Eat Disord, 2024. **57**(9): p. 1882-1889.
172. Cunningham, S.G., et al., *My Diabetes My Way: supporting online diabetes self-management: progress and analysis from 2016.* BioMedical Engineering OnLine, 2019. **18**(1): p. 13.
173. Burrichter, K., *The impact of online support groups on mental health outcomes for individuals with chronic illness.* Archives of Clinical Psychiatry, 2022. **49**(3).
174. Hixson, J.D., et al., *Patients optimizing epilepsy management via an online community: The POEM Study.* Neurology, 2015. **85**: p. 129-136.
175. Litchman, M.L., et al., *Continuous Glucose Monitoring Plus an Online Peer Support Community Reinforces Healthy Behaviors in Hispanic Adults With Type 2 Diabetes.* Diabetes Spectrum, 2022. **35**: p. 452-460.
176. Shaw, B.R., et al., *Experiences of women with breast cancer: exchanging social support over the CHESS computer network.* Journal of health communication, 2000. **5**: p. 135-159.
177. Visser, A., et al., *Group medical consultations (GMCs) and tablet-based online support group sessions in the follow-up of breast cancer: A multicenter randomized controlled trial.* Breast, 2018. **40**: p. 181-188.
178. Winzelberg, A.J., et al., *Evaluation of an internet support group for women with primary breast cancer.* Cancer, 2003. **97**: p. 1164-1173.
179. Dulli, L., et al., *A social media-based support group for youth living with HIV in Nigeria (SMART Connections): Randomized controlled trial.* Journal of Medical Internet Research Vol 22(6), 2020, ArtID e18343, 2020. **22**(6).

180. Wilford, J.G., et al., *A Multi-Modal Family Peer Support-Based Program to Improve Quality of Life among Pediatric Brain Tumor Patients: A Mixed-Methods Pilot Study.* Children, 2020. **7**(4): p. 20.
181. Geramita, E.M., et al., *The Association Between Increased Levels of Patient Engagement With an Internet Support Group and Improved Mental Health Outcomes at 6-Month Follow-Up: Post-Hoc Analyses From a Randomized Controlled Trial.* Journal of Medical Internet Research, 2018. **20**(7): p. 11.
182. Lieberman, M.A., et al., *Online support groups for Parkinson's patients: A pilot study of effectiveness.* Social Work in Health Care, 2005. **42**(2): p. 23-38.
183. Su, J., et al., *Building social identity-based groups to enhance online peer support for patients with chronic disease: a pilot study using mixed-methods evaluation.* Transl Behav Med, 2022. **12**(5): p. 702-712.
184. Huang, S.M., et al., *Developing a web-based oncofertility tool for reproductive-age women with breast cancer based on social support framework.* Supportive Care in Cancer, 2022. **30**: p. 6195-6204.
185. Nehasil, M.J., *Health-related quality of life outcomes of participants in a montana-specific, online support community for people in montana with inflammatory bowel disease.* Dissertation Abstracts International Section A: Humanities and Social Sciences, 2015. **75**(11): p. No Pagination Specified.
186. Hill, W., L. Schillo, and C. Weinert, *Effect of a computer-based intervention on social support for chronically ill rural women.* Rehabilitation Nursing Journal, 2004. **29**(5): p. 169-73.
187. Horvath, K.J., et al., *Feasibility, acceptability and preliminary efficacy of an online peer-to-peer social support ART adherence intervention.* AIDS and behavior, 2013. **17**: p. 2031-2044.
188. Glasgow, R.E., et al., *Outcomes of minimal and moderate support versions of an Internet-based diabetes self-management support program.* Journal of General Internal Medicine, 2010. **25**(12): p. 1315-1322.
189. Hull, S.J., et al., *Self-determination theory and computer-mediated support: Modeling effects on breast cancer patient's quality-of-life.* Health Communication, 2016. **31**(10): p. 1205-1214.
190. Schulz, P.J., et al., *Coping with chronic lower back pain: Designing and testing the online tool ONESELF.* Journal of Computer-Mediated Communication, 2010. **15**(4): p. 625-645.
191. Gasca, E., J. Favela, and M. Tentori, *Assisting Support Groups of Patients with Chronic Diseases through Persuasive Computing.* Journal of Universal Computer Science, 2009. **15**(16): p. 3081-3100.
192. Ghahari, S. and T. Packer, *Effectiveness of online and face-to-face fatigue self-management programmes for adults with neurological conditions.* Disability and Rehabilitation, 2012. **34**(7): p. 564-573.
193. Glasgow, R.E., et al., *Twelve-month outcomes of an Internet-based diabetes self-management support program.* Patient Education and Counseling, 2012. **87**(1): p. 81-92.
194. Han, Y., et al., *Comparison of an online versus conventional multidisciplinary collaborative weight loss programme in type 2 diabetes mellitus: A randomized controlled trial.* International Journal of Nursing Practice: p. 11.
195. Lindsay, S., et al., *The health impact of an online heart disease support group: a comparison of moderated versus unmoderated support.* Health Education Research, 2009. **24**(4): p. 646-654.
196. Merolli, M., et al. *Patient Participation in Chronic Pain Management Through Social Media: A Clinical Study*. 2016. Geneva, SWITZERLAND: Ios Press.
197. Rondags, S., et al., *Effectiveness of HypoAware, a Brief Partly Web-Based Psychoeducational Intervention for Adults With Type 1 and Insulin-Treated Type 2 Diabetes and Problematic Hypoglycemia: A Cluster Randomized Controlled Trial.* Diabetes Care, 2016. **39**(12): p. 2190-2196.

198. Wu, Q., et al., *Effects of Nurse-Led Support Via WeChat, a Smartphone Application, for Breast Cancer Patients After Surgery: A Quasi-Experimental Study.* Telemedicine journal and e-health : the official journal of the American Telemedicine Association, 2020. **26**: p. 226-234.
199. Hoybye, M.T., et al., *Effect of Internet peer-support groups on psychosocial adjustment to cancer: A randomised study.* British Journal of Cancer, 2010. **102**: p. 1348-1354.
200. Ghabrial, M.A., C.C. Classen, and J.D. Maggi, *"I've found my voice. I've found a sisterhood": Qualitative evaluation of a web-based support group for women with HIV.* Journal of HIV/AIDS & Social Services, 2020. **19**(1): p. 1-22.
201. LeBeau, K., et al., *Evaluating a novel hospital-based online health community to address palliative and psychosocial care factors for chronically ill adolescent and young adult patients.* Palliat Support Care, 2024. **22**(3): p. 432-443.
202. Graetz, I., et al., *Mobile application to support oncology patients during treatment on patient outcomes: Evidence from a randomized controlled trial.* Cancer Med, 2023. **12**(5): p. 6190-6199.
203. Yaagoob, E., et al., *WhatsApp-based intervention for people with type 2 diabetes: A randomized controlled trial.* Nursing & Health Sciences, 2024. **26**(2): p. e13117.
204. Ihrig, A., et al., *Comparison between online and face-to-face support groups for prostate cancer.* Oncology Research and Treatment, 2016. **1**: p. 133.
205. Mahlmann, S., H. Kerek-Bodden, and J. Weis, *"Courageous, colorful, active living with metastases". Scientific evaluation of online self-help meetings for women with metastatic cancer.* Onkologie: p. 6.
206. An, L.W., et al., *A Chinese pilot study of a peer support society-based smartphone wechat platform on diabetes patients' glycemic control.* Diabetes, 2016. **65**: p. A185.
207. Pretorius, C., *The experience of active involvement in an online Facebook support group, as a form of support for individuals who are diagnosed with Multiple Sclerosis.* Tydskrif Vir Geesteswetenskappe, 2016. **56**(3): p. 809-828.
208. Pester, B.D., *The efficacy of a novel facebook-based psychosocial intervention for adults with chronic pain: A randomized clinical trial.* Dissertation Abstracts International: Section B: The Sciences and Engineering, 2022. **83**(4): p. No Pagination Specified.
209. Litchman, M.L., L.S. Edelman, and G.W. Donaldson, *Effect of Diabetes Online Community Engagement on Health Indicators: Cross-Sectional Study.* JMIR Diabetes, 2018. **3**(2): p. e8.
210. Stephen, J., et al., *Evaluation of CancerChatCanada: a program of online support for Canadians affected by cancer.* Curr Oncol, 2013. **20**(1): p. 39-47.
211. Healy, E., et al., *"Whenever you need support, you first turn to the group": motivations and functions of WhatsApp groups for youth living with HIV.* AIDS Care, 2023. **35**(3): p. 437-446.
212. Tankha, H., *Engagement in a Facebook Peer support intervention and its impact on the psychosocial aspects of pain: A mixed methods investigation*, in *Psychology*. 2022, Wayne State University: Detroit.
213. Shim, M., *Self-disclosure in online support groups: The patterns of disclosure and their potential health benefits for women with breast cancer.* Dissertation Abstracts International Section A: Humanities and Social Sciences, 2009. **69**(9): p. 3379.
214. Kraan, C.F., et al., *People with arthritis, fibromyalgia and breast cancer feel empowered by their participation in online support groups.* Annals of the Rheumatic Diseases, 2006. **65**: p. 659-659.
215. Anonymous, *Online support for patients with lung cancer.* Nursing, 2009. **39**: p. 60.
216. Cox, M., et al., *Quantifying intervention engagement in a randomized controlled trial of online versus telephone-based information and support for lung cancer patients.* Asia-Pacific Journal of Clinical Oncology, 2016. **12**: p. 16.

217. Setoyama, Y., K. Nakayama, and Y. Yamazaki, *Peer support from online community on the internet among patients with breast cancer in Japan.* Studies in health technology and informatics, 2009. **146**: p. 886.
218. Kim, E., *Social support, opinion leaders, and breast cancer patients' psychosocial health outcomes in online support groups.* Dissertation Abstracts International Section A: Humanities and Social Sciences, 2013. **74**(5): p. No Pagination Specified.
219. Nam, Y., *The effect of twitter social support on health outcomes and its mediators: A randomized controlled trial of a social support intervention on twitter for patients affected by cancer.* Dissertation Abstracts International Section A: Humanities and Social Sciences, 2015. **76**(3): p. No Pagination Specified.
220. Fisher, R.E., M. Donziger, and L. Lahr, *Analysis of the MyLifeLine.org Cancer Foundation user database: Does a cancer patient website for internet communication between cancer patients and their wellwisher community assist in cancer patient support?* Journal of Clinical Oncology, 2009. **1**: p. e20700.
221. Willard, V.W., et al., *Virtual Group Activities as a New Platform for Socialization in Children With Pediatric Cancer: A Case Series From the COVID-19 Pandemic.* Journal of Pediatric Hematology/Oncology, 2022. **44**: p. 462-464.
222. Delpass and N. , *Changing the face of support: A phenomenological study of individuals with carcinoid cancer who use Facebook.* Dissertation Abstracts International: Section B: The Sciences and Engineering, 2017. **78**(2): p. No Pagination Specified.
223. Koster, E., Z. Wadhwaniya, and A.M. Namasivayam-MacDonald, *Preliminary Study of the Effects of a Dysphagia Support Group on Quality of Life.* American Journal of Speech-Language Pathology, 2023. **32**(4): p. 1466-1488.
224. Leavitt, V., et al., *Randomized controlled trial of a 12-week programme of online eSupport groups for Black and Hispanic people with multiple sclerosis.*
225. *Empowering peer-to-peer connection: Transitioning peer support from a nurse-led phone program to a peer-led multimodal online group-Increasing engagement.*
226. *Every breath: the benefits of a lung cancer support group.*
227. *De Novo Experience of Individuals with Li-Fraumeni Syndrome: Sometimes the people in your family are never going to be the ones who understand best. .*
228. *Evaluating the Efficacy of Community-Based and Online Behavioral Interventions: Experimental Design and Preliminary Insights on Health Outcomes and Obesity.*
229. *Virtual Peer Support Network Supporting Health and Wellbeing in Cardiovascular Disease Across Australia.*

Tables 8-6: Mixed Methods Appraisal Tool

Tables 8-12 display the entries for the Mixed Methods Appraisal Tool. Each table represents a different research method included within the tool: qualitative, non-randomised, randomised, descriptive, and mixed methods.

Table 8: Mixed Methods Appraisal Tool Qualitative Research

Author	Title	Are there clear research questions?	Do the collected data allow to address the research question?	Is the qualitative approach appropriate to answer the research question?	Are the qualitative data collection methods adequate to address the research question?	Are the findings adequately derived from the data?	Is the interpretation of results sufficiently substantiated by data?	Is there coherence between qualitative data sources, collection, analysis, and interpretation?
Shoebotham and Coulson	Therapeutic Affordances of Online Support Group Use in Women With Endometriosis	Yes	Yes	Yes	Can't tell	Yes	Yes	Yes
Russell et al	Support amid uncertainty: Long COVID illness experiences and the role of online communities	Yes	Yes	Yes	Yes	Yes	Yes	Yes
Bazrafshani et al	The role of online social networks in	Yes	Yes	Yes	Can't tell	Can't tell	Yes	Yes

	improving health literacy and medication adherence among people living with HIV/AIDS in Iran: Development of a conceptual model							
Day	Exploring Online Peer Support Groups for Adults Experiencing Long COVID in the United Kingdom: Qualitative Interview Study	Yes	No	Yes	No	Yes	Yes	Yes
Willis et al	The Power of Peers: Applying User-Generated Content to Health Behaviors “Off-Line”	Yes	Can't tell	Yes	Can't tell	Yes	Yes	Yes
Mo and Coulson	Are online support groups always beneficial? A qualitative exploration of the empowering	Yes	Yes	Yes	Yes	Yes	Yes	Yes

	and disempowering processes of participation within HIV/AIDS-related online support groups							
Healy et al	“Whenever you need support, you first turn to the group”: motivations and functions of whatsapp groups for youth living with HIV	Yes	Yes	Yes	No	Yes	Yes	Yes
Van Uden Kraan et al	Empowering Processes and Outcomes of Participation in Online Support Groups for Patients With Breast Cancer, Arthritis, or Fibromyalgia	Yes	Can't tell	Yes	Yes	No	Yes	Yes
Holbrey and Coulson	A qualitative investigation of the impact of peer to peer online support for women living with Polycystic	Yes	Yes	Yes	Yes	Yes	Yes	Yes

	Ovary Syndrome							
Litchman	A multiple method analysis of peer health in the diabetes online community - chapter 5	Yes	Yes	Yes	Yes	Yes	Yes	Yes
Steadman and Pistorius	The impact of an online Facebook support group for people with multiple sclerosis on non-active users	Yes	Yes	Yes	Yes	Yes	Yes	Yes
Zigron and Bronstein	“Help is where you find it”: The role of weak ties networks as sources of information and support in virtual health communities	Yes	Yes	Yes	Yes	Yes	Yes	Yes
Ashtari and Taylor	The Internet Knows More Than My Physician: Qualitative Interview Study of People With Rare Diseases	Yes	Yes	Yes	Yes	Yes	Yes	Yes

	and How They Use Online Support Groups							
Brady et al	Accessing support and empowerment online: The experiences of individuals with diabetes	No	Can't tell	Yes	Yes	Yes	Yes	Yes
Wilson and Stock	'Social media comes with good and bad sides, doesn't it?' A balancing act of the benefits and risks of social media use by young adults with long-term conditions	Yes	Yes	Yes	Yes	Yes	Yes	Yes
Coulson	How do online patient support communities affect the experience of inflammatory bowel disease? An online survey	No	Yes	Yes	No	Yes	Yes	Yes
Iliffe and Thompson	Investigating the beneficial experiences of online peer	Yes	Yes	Yes	Yes	Yes	Yes	Yes

	support for those affected by alopecia: an interpretative phenomenological analysis using online interviews							
Zhu et al	Mobile Breast Cancer e-Support Program for Chinese Women With Breast Cancer Undergoing Chemotherapy (Part 1): Qualitative Study of Women's Perceptions	Yes	Yes	Yes	Yes	Yes	Yes	Yes
Willis	Applying the Health Belief Model to Medication Adherence: The Role of Online Health Communities and Peer Reviews	Yes	Can't tell	Yes	Yes	Yes	Yes	Yes
Vilhauer	Perceived benefits of online support	No	N/A	Yes	Yes	No	Yes	Yes

	groups for women with metastatic breast cancer							
Egerton	Expert-Moderated Peer-to-Peer Online Support Group for People With Knee Osteoarthritis: Mixed Methods Randomized Controlled Pilot and Feasibility Study	Yes	Yes	Yes	Yes	Yes	Yes	Yes
Meade	Members' Experiences of a Neuromuscular Disorder Online Support Group	Yes	Yes	Yes	No	Yes	Yes	Yes
Rowlands et al	A qualitative exploration of the psychosocial needs of people living with long-term conditions and their perspectives on online peer support	Yes	Yes	Yes	Yes	Yes	Yes	Yes

Parrocha and Bernadas	‘They Know What It’s Like’: Exploring Facebook Groups for Digital Coping	Yes	Yes	Yes	Yes	Yes	Yes	Yes
Mackie	Finding my tribe: a qualitative interview study of how people living with metastatic breast cancer perceive support groups	Yes	Yes	Yes	Yes	Yes	Yes	Yes
Walsh	“Living with Loss”: A qualitative exploration of existential fears among people with advanced lung cancer in online lung cancer support groups	Yes	Yes	Yes	Yes	Yes	Yes	Yes
Mills	Online support groups, social identity, and the health and wellbeing of adults with Long	Yes	Yes	Yes	Yes	Yes	Yes	Yes

	Covid: An interview study							
Hurtado	Towards quality of life: experiences and health-behavior change in chronic and oncological patients in virtual communities	Yes	Yes	Yes	Can't tell	No	No	No
Hodson and O'Meara	Curating Hope: The Aspirational Self and Social Engagement in Early-Onset Cancer Communities on Social Media	No	No	No	Can't tell	Yes	Yes	Yes
Garrett	The Role of Social Media in the Experiences of COVID-19 Among Long-Hauler Women: Qualitative Study	No	No	No	Can't tell	Yes	Yes	Yes

Table 9: MMAT Non-Randomised Research

Author	Title	Are there clear research questions?	Do the collected data allow to address the research question?	Are the participants representative of the target population?	Are the measurements appropriate regarding both the outcome and intervention?	Are there complete outcome data?	Are the confounders accounted for in the design and analysis?	During the study period, is the intervention administered (or exposure) as intended?
Huber et al	Face-to-face vs. online peer support groups for prostate cancer: A cross-sectional comparison study	Yes	Yes	Can't tell	Yes	Yes	Can't tell	Can't tell
Lieberman et al	Electronic support groups for breast carcinoma	Yes	Yes	Yes	Yes	Yes	No	Yes
Han et al	Lurking as an Active Participation Process: A Longitudinal Investigation of Engagement with an Online Cancer Support Group	Yes	Yes	Can't tell	Yes	Can't tell	Yes	Yes
Shaw et al	Communicating about self and others within an online support group for women with breast cancer and subsequent outcomes	Yes	Yes	Can't tell	Yes	Can't tell	Yes	No

Shim et al	How Does Insightful and Emotional Disclosure Bring Potential Health Benefits?: Study Based on Online Support Groups for Women with Breast Cancer	Yes	Yes	Can't tell	Yes	Can't tell	Yes	Yes
Kim et al	The process and effect of supportive message expression and reception in online breast cancer support groups	Yes	Yes	Can't tell	Yes	Can't tell	Yes	Yes
Shaw et al	Effects of prayer and religious expression within computer support groups on women with breast cancer	Yes	Yes	Can't tell	Yes	Can't tell	Yes	No
Shaw et al	Effects of Insightful Disclosure Within Computer	Yes	Yes	Can't tell	Yes	Can't tell	Yes	No
Han et al	A Longitudinal Investigation of Empathic Exchanges in Online Cancer Support Groups: Message Reception and Expression Effects on Patients' Psychosocial Health Outcomes	Yes	Yes	Can't tell	Yes	Yes	Yes	Yes

Kim et al	Opinion Leaders in Online Cancer Support Groups: An Investigation of Their Antecedents and Consequences	Yes	Yes	Can't tell	Yes	Can't tell	Yes	Can't tell
Leavitt et al	eSupport: Feasibility trial of telehealth support group participation to reduce loneliness in multiple sclerosis	No	Can't tell	Can't tell	Yes	Can't tell	No	Yes
Costello et al	Associations Between Engagement With an Online Health Community and Changes in Patient Activation and Health Care Utilization: Longitudinal Web-Based Survey	Yes	Yes	No	Yes	Can't tell	No	Can't tell
Letourneau et al	Impact of Online Support for Youth With Asthma and Allergies: Pilot Study	Yes	Yes	Can't tell	Yes	No	No	Can't tell
Batenburg and Das	Emotional Approach Coping and the Effects of Online Peer-Led Support Group Participation Among Patients With Breast Cancer: A Longitudinal Study	Yes	Yes	No	Yes	Can't tell	Yes	Can't tell

Lieberman et al	Electronic support groups for breast carcinoma	Yes	No		Yes	Yes	No	Yes
Lange et al	Effectiveness, acceptance and satisfaction of guided chat groups in psychosocial aftercare for outpatients with prostate cancer after prostatectomy	Yes	Yes	No	Yes	Yes	Yes	Yes
Lieberman and Winzelberg	The relationship between religious expression and outcomes in online support groups: A partial replication	Yes	Yes	Can't tell	Yes	Can't tell	Yes	Yes
Petrovski and Zivkovic	Are We Ready to Treat Our Diabetes Patients Using Social Media? Yes, We Are	Yes	No	Can't tell	Yes	Can't tell	No	Yes
Ronen	Facilitated WhatsApp Support Groups for Youth Living With HIV in Nairobi, Kenya: Single-Arm Pilot Intervention Study	No	No	Can't tell	Yes	No	No	Can't tell

Table 10: MMAT Randomised Studies

Author	Title	Are there clear research questions?	Do the collected data allow to address the research question?	Is randomisation appropriately performed?	Are the groups comparable at baseline?	Are there complete outcome data?	Are outcome assessors blinded to the intervention provided?	Did the participants adhere to the assigned intervention?
Pester et al	Facing Pain Together: A Randomized Controlled Trial of the Effects of Facebook Support Groups on Adults With Chronic Pain	Yes	No	Yes	Yes	Yes	Can't tell	Yes
Tankha	Engagement in a Facebook peer support intervention and its impact on the psychosocial aspects of pain: a mixed methods investigation	Yes	No	Yes	Can't tell	Yes	Can't tell	Yes
Lopez-Olivo	A randomized controlled trial evaluating the effects of social networking on chronic disease management in rheumatoid arthritis	No	Can't tell	Yes	Yes	Can't tell	Yes	Yes
Baydoun et al	Comparing online support groups with psychoeducation versus psychoeducation alone for distressed breast cancer survivors: a randomized controlled trial	Yes	Yes	Yes	Yes	Yes	Can't tell	Can't tell
Koufopolous et al	A Web-Based and Mobile Health Social Support Intervention to Promote Adherence to Inhaled Asthma Medications: Randomized Controlled Trial	Yes	Yes	Yes	Yes	Yes	No	No

Changrani	Online cancer support groups: experiences with underserved immigrant	Yes	Yes	Can't tell	Can't tell	Can't tell	Can't tell	Yes
Salzer	A randomized, controlled study of Internet peer-to-peer interactions among women newly diagnosed with breast cancer	Yes	Yes	Can't tell	Yes	Can't tell	Can't tell	Yes

Table 11: MMAT Descriptive studies

Author	Title	Are there clear research questions?	Do the collected data allow to address the research question?	Is the sampling strategy relevant to address the research question?	Is the sample representative of the target population?	Are the measurements appropriate?	Is the risk of nonresponse bias low?	Is the statistical analysis appropriate to answer the research question?
Herrero et al	Participation of Patients With Type 2 Diabetes in Online Support Groups is Correlated to Lower Levels of Diabetes Self-Management	Yes	No	Yes	No	Yes	Can't tell	No
Bazrafshani et al	The role of online social networks in improving health literacy and medication adherence among people living with HIV/AIDS in Iran: Development of a conceptual model	No	No	No	Can't tell	Yes	No	No
Fullwood et al	Lurking towards empowerment: Explaining propensity to engage with online health support groups and its association with positive outcomes	Yes	Yes	Can't tell	Can't tell	Yes	Can't tell	Yes
Babyar	The role of social media in the relationship between social support and adherence in children with cystic fibrosis	Yes	Yes	Yes	Yes	Yes	Can't tell	Can't tell

Beaudoin and Tao	Benefiting from social capital in online support groups: an empirical study of cancer patients	Yes	Can't tell	Yes	Can't tell	Yes	Can't tell	Yes
Mo and Coulson	Developing a model for online support group use, empowering processes and psychosocial outcomes for individuals living with HIV/AIDS	Yes	Can't tell	Yes	Can't tell	Yes	Can't tell	Yes
Lieberman et al	The role of insightful disclosure in outcomes for women in peer-directed breast cancer groups: A replication study	Yes	Yes	Yes	Can't tell	Yes	Can't tell	Yes
Mo and Coulson	Living with HIV/AIDS and Use of Online Support Group	yes	Yes	Yes	No	Yes	Can't tell	Yes
Seçkin	I Am Proud and Hopeful: Age-Based Comparisons in Positive Coping Affect Among Women Who Use Online Peer-Support	Yes	Yes	No	No	Can't tell	Can't tell	Yes
van Uden Kraan et al	Participation in online patient support groups endorses patients’ empowerment	Yes	Yes	Yes	No	No	Can't tell	Yes
Hansen	Lifestyle changes among people with type 2 diabetes are associated with participation in online groups and time since diagnosis	Yes	Yes	Yes	No	Yes	No	Yes

Chung et al	Perceived online social support for Parkinson's disease patients: The role of support type, uncertainty, contentment, and psychological quality of life	Yes	Yes	Yes	No	Yes	No	Yes
Chen et al	Interrelationship between Interpersonal Interaction Intensity and Health Self-Efficacy in People with Diabetes or Prediabetes on Online Diabetes Social Platforms: An In-Depth Survey in China	Yes	Yes	Yes	Can't tell	Yes	Can't tell	Yes
Huber et al	The effect of an online support group on patients׳ treatment decisions for localized prostate cancer: An online survey	Yes	Yes	Yes	Can't tell	Yes	No	Yes
Setoyama	Benefits of Peer Support in Online Japanese Breast Cancer Communities: Differences Between Lurkers and Posters	Yes	Yes	Yes	Can't tell	Yes	No	Yes
Kosugi	Association Between Loneliness and the Frequency of Using Online Peer Support Groups Among Cancer Patients With Minor Children: A Cross-Sectional Web-Based Study	Yes - aim	Yes	No	Can't tell	Yes	Can't tell	Yes
Cummings et al	Beyond hearing: Where real-world and online support meet	Yes	Yes	No	Can't tell	No	No	No

Zheng et al	Improving customer well-being through two-way online social support	Yes	Yes	Yes	No	Yes	Yes	Yes
Parrish et al	Perceptions of the members of an online support group for women with gynecologic cancers and pre-cancers regarding online support, illness, information, and awareness	Yes	Yes	Yes	Can't tell	No	Yes	No
Mo and Coulson	Online support group use and psychological health for individuals living with HIV/AIDS	Yes	Yes	Yes	Can't tell	Yes	Can't tell	Yes
Sparling et al	In-person and online social participation and emotional health in individuals with multiple sclerosis	Yes	Yes	Yes	No	Yes	Can't tell	Yes
Algtewi et al	Online support groups for head and neck cancer and health-related quality of life	Yes	Yes	Yes	Can't tell	Yes	Can't tell	Yes
Seckin	Psychological support using internet: Who joins online cancer support groups and patterns of participation	Yes	Can't tell	Yes	Can't tell	No	Can't tell	No
Batenburg and Dase	Emotional Coping Differences Among Breast Cancer Patients From an Online Support Group: A Cross-Sectional Study	Yes	Yes	Yes	Can't tell	Yes	Can't tell	Yes

van Uden Kraan et al	Self-Reported Differences in Empowerment Between Lurkers and Posters in Online Patient Support Groups	Yes	Can't tell	Yes	Can't tell	Yes	Can't tell	Yes
Batenburg and Dase	Virtual Support Communities and Psychological Well-Being: the Role of Optimistic and Pessimistic Social Comparison Strategies	Yes	Yes	Yes	Can't tell	Yes	Can't tell	Yes
Bartlett and Coulson	An investigation into the empowerment effects of using online support groups and how this affects health professional/patient communication	Yes	Yes	Yes	Can't tell	Can't tell	Can't tell	Yes
Kaal et al	Online support community for adolescents and young adults (AYAs) with cancer: user statistics, evaluation, and content analysis	Yes	Yes	Yes	Can't tell	Can't tell	Can't tell	Yes
Herrero et al	Do online support groups influence the well-being of patients with diabetes?	Yes	Yes	No	Can't tell	No	Can't tell	Yes
Meng et al	How Cancer Patients Benefit from Support Networks Offline and Online: Extending the Model of Structural-to-Functional Support	Yes	Yes	Yes	No	Yes	Can't tell	Yes

Mo and Coulson	Empowering processes in online support groups among people living with HIV/AIDS: A comparative analysis of 'lurkers' and 'posters'	Yes	Yes	Yes	No	Yes	Can't tell	Yes
Morehouse et al	Impacts of online support groups on quality of life, and perceived anxiety and depression in those with ME/CFS: a survey	Yes	Yes	Yes	Yes	Can't tell	Can't tell	Yes
Vanstrumet al	An Exploration of Online Support Community Participation Among Patients With Vestibular Disorders	Yes	Yes	No	Can't tell	No	Can't tell	Yes
Litchman	A multiple method analysis of peer health in the diabetes online community - chapter 4	Yes	Yes	Yes	Can't tell	No	Can't tell	Yes
Thewlis	The Role of Online Social Support for Individuals Living with Disabilities and Chronic Illness: Investigating Stress, Resilience and Positive Mental Health	Yes	No	Yes	Can't tell	No	No	No

Terborg	Managing a Chronic Illness: An Exploratory Cross-sectional Study on the Association Between Contributing to an Online Self-help Platform and the Self-management, Illness Perception, and Chronic Care Assessment of Users Suffering From Chronic Tinnitus	Yes	Yes	Yes	Can't tell	Yes	No	Yes
Vanstrumet al	Utilization of Face-to-Face Vestibular Support Groups: A Comparison to Online Group Participation	Yes	Can't tell	Yes	Can't tell	Can't tell	No	Yes
Tam et al	Head and Neck Cancer Online Support Groups: Disparities in Participation and Impact on Patients	No	N/A	Yes	Can't tell	Can't tell	No	No
Rose et al	Characterization of the role of Facebook groups for patients who use scalp cooling therapy: a survey study	No	N/A	N/A	Can't tell	Yes	No	No

Table 12: MMAT Mixed Methods Studies

Authors	Title	Are there clear research questions	Do the collected data allow to address the research questions	Is there an adequate rationale for using a mixed methods design to address the research question?	Are the different components of the study effectively integrated to answer the research question?	Are the outputs of the integration of qualitative and quantitative components adequately interpreted?	Are divergences and inconsistencies between quantitative and qualitative results adequately addressed?	Do the different components of the study adhere to the quality criteria of each tradition of the methods involved?
Yao et al	The Impact of Online Social Support on Patients' Quality of Life and the Moderating Role of Social Exclusion	Yes	Yes	Yes	No	No	No	Yes
Stewart et al	Impacts of online peer support for children with asthma and allergies: It just helps you every time you can't breathe well"	Yes	Yes	No	Yes	Yes	No	Yes
Kever et al	Feasibility trial of a telehealth support group intervention to reduce anxiety in multiple sclerosis	Yes	Yes	No	No	No	No	No
Cooper et al	Social media support group: Implementation and evaluation	No	Yes	No	Yes	Yes	No	No

Vilhauer	Perceived Benefits of Online Support Groups for Women with Metastatic Breast Cancer	Yes	Yes	No	No	Yes	No	Yes
Litchman	A multiple method analysis of peer health in the diabetes online community - Chapter 6	Yes	Yes	No	No	Yes	No	Yes
Bazrafshani et al	The role of online social networks in improving health literacy and medication adherence among people living with HIV/AIDS in Iran: Development of a conceptual model	Yes	Yes	Yes	Yes	Yes	No	Yes
Holdren	Perspectives of People With Cancer or Hereditary Cancer Risk on the Use and Value of Online Peer Support	Yes	Can't tell	No	Yes	Yes	No	No
Ashtari and Taylor	Patients With Rare Diseases and the Power of Online Support Groups: Implications for the Medical Community	No	Can't tell	No	No	Yes	No	No
