# Supplementary material for: A mixed studies systematic review on the health and wellbeing effects, and underlying mechanisms, of online support groups for chronic conditions
Source: Commun Psychol. 2025 Mar 15;3:40. doi: 10.1038/s44271-025-00217-6 (PMC11910600; doi:10.1038/s44271-025-00217-6)
Supplement: Supplementary file 3 — Reporting summary [file 44271_2025_217_MOESM3_ESM.pdf]

nature portfolio

Corresponding author(s): Freya Mills

Last updated by author(s): 23/01/2025

Reporting Summary

Nature Portfolio wishes to improve the reproducibility of the work that we publish. This form provides structure for consistency and transparency in reporting. For further information on Nature Portfolio policies, see our Editorial Policies and the Editorial Policy Checklist.

Statistics

For all statistical analyses, confirm that the following items are present in the figure legend, table legend, main text, or Methods section.

n/a	Confirmed	
☒	☐	The exact sample size (*n*) for each experimental group/condition, given as a discrete number and unit of measurement
☒	☐	A statement on whether measurements were taken from distinct samples or whether the same sample was measured repeatedly
☒	☐	The statistical test(s) used AND whether they are one- or two-sided<br>*Only common tests should be described solely by name; describe more complex techniques in the Methods section.*
☒	☐	A description of all covariates tested
☒	☐	A description of any assumptions or corrections, such as tests of normality and adjustment for multiple comparisons
☒	☐	A full description of the statistical parameters including central tendency (e.g. means) or other basic estimates (e.g. regression coefficient) AND variation (e.g. standard deviation) or associated estimates of uncertainty (e.g. confidence intervals)
☒	☐	For null hypothesis testing, the test statistic (e.g. *F*, *t*, *r*) with confidence intervals, effect sizes, degrees of freedom and *P* value noted<br>*Give P values as exact values whenever suitable.*
☒	☐	For Bayesian analysis, information on the choice of priors and Markov chain Monte Carlo settings
☒	☐	For hierarchical and complex designs, identification of the appropriate level for tests and full reporting of outcomes
☒	☐	Estimates of effect sizes (e.g. Cohen's *d*, Pearson's *r*), indicating how they were calculated

Our web collection on statistics for biologists contains articles on many of the points above.

Software and code

Policy information about availability of computer code

Data collection	Rayyan was used for screening articles. Mourad Ouzzani, Hossam Hammady, Zbys Fedorowicz, and Ahmed Elmagarmid. Rayyan — a web and mobile app for systematic reviews. Systematic Reviews (2016) 5:210, DOI: 10.1186/s13643-016-0384-4.
Data analysis	No software was used for the analysis

For manuscripts utilizing custom algorithms or software that are central to the research but not yet described in published literature, software must be made available to editors and reviewers. We strongly encourage code deposition in a community repository (e.g. GitHub). See the Nature Portfolio guidelines for submitting code & software for further information.

Data

Policy information about availability of data

All manuscripts must include a data availability statement. This statement should provide the following information, where applicable:

- Accession codes, unique identifiers, or web links for publicly available datasets
- A description of any restrictions on data availability
- For clinical datasets or third party data, please ensure that the statement adheres to our policy

Data and materials used for this review are available within the text and in the supplementary files.

Research involving human participants, their data, or biological material

Policy information about studies with human participants or human data. See also policy information about sex, gender (identity/presentation), and sexual orientation and race, ethnicity and racism.

Reporting on sex and gender	No data from participants was collected for this paper as this was a systematic review. Gender and sex aren't described within the context of the findings of this review.
Reporting on race, ethnicity, or other socially relevant groupings	No data from participants was collected for this paper as this was a systematic review. Race, ethnicity and other socially relevant groupings aren't described within the context of the findings of this review.
Population characteristics	The participants of the included studies were mostly from the United States of America and United Kingdom.
Recruitment	No participants were recruited as this was a systematic review. Participants of the included studies were mostly recruited from existing online support groups, or hospitals or healthcare facilities. The studies are at risk of self-selection bias as the participants are more likely to have stronger opinions on the use of online support groups and/or are more likely to have less severe symptoms than those not taking part.
Ethics oversight	As this was a systematic review and no primary data was collected, ethical approval was not needed for this study.

Note that full information on the approval of the study protocol must also be provided in the manuscript.

Field-specific reporting

Please select the one below that is the best fit for your research. If you are not sure, read the appropriate sections before making your selection.

☐ Life sciences ☒ Behavioural & social sciences ☐ Ecological, evolutionary & environmental sciences

For a reference copy of the document with all sections, see nature.com/documents/nr-reporting-summary-flat.pdf

Behavioural & social sciences study design

All studies must disclose on these points even when the disclosure is negative.

Study description	This is a systematic review exploring the effects of online support groups for chronic conditions on the health and wellbeing of group members. This review includes quantitative, qualitative, and mixed methods studies and the results are written in a thematic syntheis. Four databases were searched, as well as pre-publication databases and grey literature searches.
Research sample	Most studies were conducted in the USA or UK. Most studies included participants who had cancer, HIV or included various long-term conditions. Samples sizes ranged from 6 to 1641.
Sampling strategy	Studies were included if they met the inclusion criteria of: i) exploring any type of online support group; ii) included participants with a chronic physical health condition; and iii) included a health or wellbeing outcome. Within the included studies, most participants were recruited via online support groups or hospitals / healthcare facilities.
Data collection	Data were collected from four online databases, pre-publication databases and grey literature searches. Data were screened in Rayyan. The third author screened 20% of title and abstracts and screened all of the excluded studies.
Timing	The searched included studies from the origin of the database to September 11th 2024. For pre-publication databases, Google Scholar and Google Advanced Search the first / most relevant 200 articles were considered.
Data exclusions	Studies were excluded if they did not meet the following criteria: participants did not have a chronic physical health condition (e.g., caregivers or those with a mental health condition); the study (or its measures) did not assess online support groups; the outcomes were not related to health or wellbeing (e.g., acceptability of online support groups); the papers were not published in English; the papers were an abstract or review; the paper did not include primary data (e.g., analysed only the content of the support group)
Non-participation	Some of the experimental studies discussed non-participation. As many studies were cross-sectional and recruiting via online support groups with an unknown active audience, they were unable to identify non-participation rates.
Randomization	Some of the experimental studies included in the review randomised participants to the online support group or control (e.g., waitlist or education)

Reporting for specific materials, systems and methods

We require information from authors about some types of materials, experimental systems and methods used in many studies. Here, indicate whether each material, system or method listed is relevant to your study. If you are not sure if a list item applies to your research, read the appropriate section before selecting a response.

Materials & experimental systems

n/a	Involved in the study
☒	☐ Antibodies
☒	☐ Eukaryotic cell lines
☒	☐ Palaeontology and archaeology
☒	☐ Animals and other organisms
☒	☐ Clinical data
☒	☐ Dual use research of concern
☒	☐ Plants

Methods

n/a	Involved in the study
☒	☐ ChIP-seq
☒	☐ Flow cytometry
☒	☐ MRI-based neuroimaging

Plants

Seed stocks

Report on the source of all seed stocks or other plant material used. If applicable, state the seed stock centre and catalogue number. If plant specimens were collected from the field, describe the collection location, date and sampling procedures.

Novel plant genotypes

Describe the methods by which all novel plant genotypes were produced. This includes those generated by transgenic approaches, gene editing, chemical/radiation-based mutagenesis and hybridization. For transgenic lines, describe the transformation method, the number of independent lines analyzed and the generation upon which experiments were performed. For gene-edited lines, describe the editor used, the endogenous sequence targeted for editing, the targeting guide RNA sequence (if applicable) and how the editor was applied.

Authentication

Describe any authentication procedures for each seed stock used or novel genotype generated. Describe any experiments used to assess the effect of a mutation and, where applicable, how potential secondary effects (e.g. second site T-DNA insertions, mosiacism, off-target gene editing) were examined.
